# Complement activation by autoantigen recognition in the growth process of benign prostatic hyperplasia

**DOI:** 10.1038/s41598-019-57001-w

**Published:** 2019-12-30

**Authors:** Junya Hata, Takeshi Machida, Kanako Matsuoka, Seiji Hoshi, Hidenori Akaihata, Hiroyuki Hiraki, Toshiyuki Suzuki, Soichiro Ogawa, Masao Kataoka, Nobuhiro Haga, Kei Ishibashi, Yoshimi Homma, Hideharu Sekine, Yoshiyuki Kojima

**Affiliations:** 1 Department of Urology, Fukushima Medical University School of Medicine, Fukushima, 960-1295 Japan; 2 Department of Immunology, Fukushima Medical University School of qwMedicine, Fukushima, 960-1295 Japan; 3 Department of Biomolecular Science Institute of Biomedical Sciences, Fukushima Medical University School of Medicine, Fukushima, 960-1295 Japan

**Keywords:** Complement cascade, Prostate

## Abstract

The pathophysiology of benign prostatic hyperplasia (BPH) remained unclear. Here, we concentrated on the complement activation in the growth of BPH using a rat model. BPH tissues were harvested from rats after rat urogenital sinus implantation. The local expression and deposition levels of C1q, C3, mannose-binding lectin (MBL), factor B (FB), and C5b-9 in the rat and human BPH tissues were analyzed by real-time RT-PCR, western blotting and immunohistochemistry (IHC). Serum IgG levels in the rat BPH model were analyzed by ELISA, and IHC was used to assess tissue localization. Proteins binding serum IgG autoantibody in the BPH rats were isolated by immunoprecipitation. C1q, C3, MBL, FB and C5b-9 were highly localized in rat BPH tissues compared to normal tissues. In contrast, C3, FB and C5b-9, but not C1q and MBL, were abundantly detected in human BPH tissues compared to normal tissues. Diffuse localization of IgG in rat BPH tissues was found. Heat shock protein 90, annexin, α-smooth muscle actin, and β-actin were identified as targets for IgG autoantibodies in the BPH model. Our results strongly suggested the role for complement activation in the growth process of BPH, likely triggered by classical pathway activation with autoantibodies.

## Introduction

Benign prostatic hyperplasia (BPH) involves histological changes and hyperplasia of stromal and epithelial cells within discrete nodules that are generally located in the transition zone of the prostate^1^. Although androgens are involved in causing BPH, androgens alone cannot be cited as the cause for all cases of BPH. In recent studies, inflammation has attracted attention as a non-androgen pathway for the development of BPH^2^. The cause of inflammation could include infection, mechanical stimulation, hormonal change, and autoimmunity^3–5^. However, how these factors are responsible for the pathogenesis of BPH remains unclear.

The complement system is a major component of the innate immune system, is activated via the classical, lectin or alternative pathways, and is involved in defending against foreign pathogens through complement fragments that participate in opsonization, chemotaxis, and activation of leukocytes and through cytolysis by the terminal complement complex (C5b-9)^6^. Complement activation also participates in the clearance of apoptotic cells and immune complexes. In addition to these well-appreciated properties, the complement system plays an integrative role in the regulation of innate and adaptive immune responses and is involved in tissue regeneration, angiogenesis, and amplification of inflammation^7^. However, uncontrolled activation of the complement system can cause tissue injury, and there is clear evidence that the complement pathway is activated in many inflammatory diseases including infection and ischemia-reperfusion injury, and autoimmune diseases such as systemic lupus erythematosus^8^.

In a previous study, we performed a DNA microarray analysis using stromal-dominant BPH model rats, which have characteristics that mimic the growth process of human BPH, with a view to elucidating the mechanism of BPH^9–11^. The rat BPH model was generated by implantation of the urogenital sinus (UGS) from male rat 20-day embryos to the prostate tissues of pubertal male rats, in which the implanted UGS developed into the BPH-like tissue time-dependently. The rat BPH tissue was subjected to whole genome expression analysis and compared to the normal prostate tissue of the same rat. Functional network analysis in the study showed that the gene expression of *C1qa*, *C1qb*, *C1qc*, *C1r*, and *C2*, the complement components of the classical pathway, were significantly increased, and the expression of *Cd59*, the major cell surface inhibitor of C5b-9 formation, was significantly decreased in the BPH tissue of the BPH rat model compared to their normal prostate tissue^9^. On the other hands, the classical complement pathway was reported to be associated with the suppression or progression of various cancers^12–14^. These results and reports suggested that the role of complement activation via the classical pathway in the growth process of BPH in this model should be clarified.

To clarify the involvement of complement activation in the mechanism of BPH growth, we analyzed the expression and deposition of complement components, including the classical pathway, in prostate tissue of BPH model rats and BPH patients.

## Results

### Complement expression and deposition in prostate tissues of the rat BPH model

**Figure 1:**
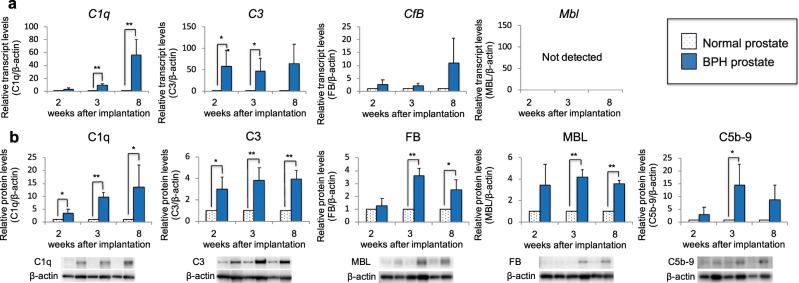
Expression and deposition levels of complement factors in BPH-like tissues of the rat BPH model. The left ventral prostate was surgically excised from rats that had received UGS implantation and was used as the BPH tissue. The right prostate free from the BPH lesion was excised separately from the same rat and used as the normal prostate tissue. (**a**) mRNA expression levels for complement factors in BPH and normal prostate tissues were analyzed by qRT-PCR. (**b**) Protein levels, representing local expression and deposition of complement factors in the BPH and normal prostate tissues, were analyzed by western blotting. Data are shown as means ± SEM (*n* = 4). *p < 0.05, **p < 0.01.

On the basis of our previous DNA microarray findings using the BPH model rat (Supplementary Table S1), we performed qRT-PCR to investigate the mRNA expression levels of complement components including *C1q*, *C3*, *Mbl*, and *Cfb*. As shown in Fig. 1a, the expression levels of *C1q* were higher in rat BPH tissues than controls at 2, 3, and 8 weeks after UGS implantation, with statistical significance observed at 3 and 8 weeks. The expression levels of *C3* were also higher in the rat BPH tissues than controls throughout the testing period, with statistical significance observed at 2 and 3 weeks. The expression levels of *Cfb* were also higher in the rat BPH tissues than controls; however, statistical significance was not reached at any time point tested. The expression levels of *Mbl* were undetectable in both the rat BPH and control tissues throughout the testing period. The mRNA expression levels of C5b-9, the terminal pathway complex, were not analyzed since it is a protein complex composed of several different complement factors.

Next, we measured protein levels of C1q, C3, MBL, and FB in prostate tissue extracts from BPH rats by western blotting. Similar to the mRNA expression results, the protein levels of C1q and C3 were higher in the extracts from BPH rats than controls, with statistical significance observed at the earliest period examined (2 weeks), and were maintained at high levels throughout the testing period (Fig. 1b). The protein levels of MBL and FB were also higher in the extracts from BPH rats than controls, with statistical significance observed at 3 and 8 weeks. C5b-9 protein levels were also higher in the extracts from BPH rats than controls, with statistical significance observed at 3 weeks.

**Figure 2:**
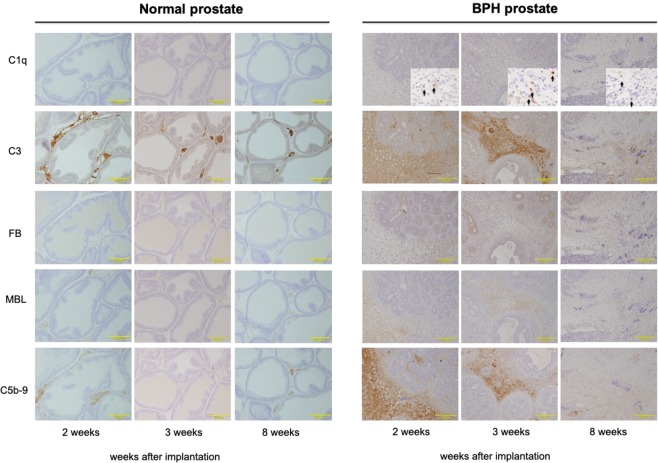
Detection of complement factors by immunohistochemical analysis of BPH-like tissues of the rat BPH model. Expression and deposition levels of complement factors in prostate tissues were analyzed by immunohistochemistry with paraffin-embedded prostate sections and corresponding antibodies. Similar results were obtained from 4 individual rats; representative images for each group are shown (Magnification, ×200).

We further examined the localization of complement components in prostate tissues of the BPH rats using immunohistochemical analysis. As shown in Fig. 2, rat BPH tissues has an increase of fibrous components time-dependently (from 2 to 8 weeks) in stromal area of rat BPH tissues. C1q was sporadically detected in cells in the stromal area at 2 weeks after UGS implantation, suggesting local expression of C1q in the rat BPH tissue, and the frequency of C1q-positive cells was increased at 3 weeks, which is consistent with the expression results. C3 was strongly detected in stromal-dominant areas and areas with some stromal cells at 2 and 3 weeks after UGS implantation, suggesting both local expression and deposition of C3 in the rat BPH tissue. FB was detected strongly in epithelial-dominant areas and sporadically in cells at 2 and 3 weeks after UGS implantation, suggesting deposition in the rat BPH tissue is more dominant than its local expression. MBL and C5b-9 were strongly detected in stromal-dominant areas and areas with some stromal cells at 2 and 3 weeks. Detected levels of the complement proteins, especially in C1q and MBL, were decreased at 8 weeks compared to levels observed at 2 and 3 weeks, which is likely due to an increase in fibrous components in BPH tissue. In contrast, those proteins were absent or detected at minimal levels in normal prostate tissues at all time points tested (Fig. 2).

In addition, we measured serum C3 levels in rat sera from BPH and intact rats free from surgical UGS implantation by ELISA. This result showed that BPH rats had significantly increased serum C3 levels compared to intact rats (98.0 μg/mL vs. 168.9 μg/mL; *p* < 0.05) (Supplementary Fig. S3). These results suggest significant involvement of the complement pathway in the growth process of BPH in this model.

Taken together, our results strongly suggest that the complement system is involved in the growth of BPH tissue lesions in the rat BPH model. Our results also suggest that the growth of BPH lesions in rat BPH tissue was initiated by activation of the classical pathway by the binding of C1q to antigen-antibody complexes, with subsequent activation of the lectin pathway (by the binding of MBL) and the alternative pathway.

### Complement expression and deposition in prostate tissues of human BPH patients

**Figure 3:**
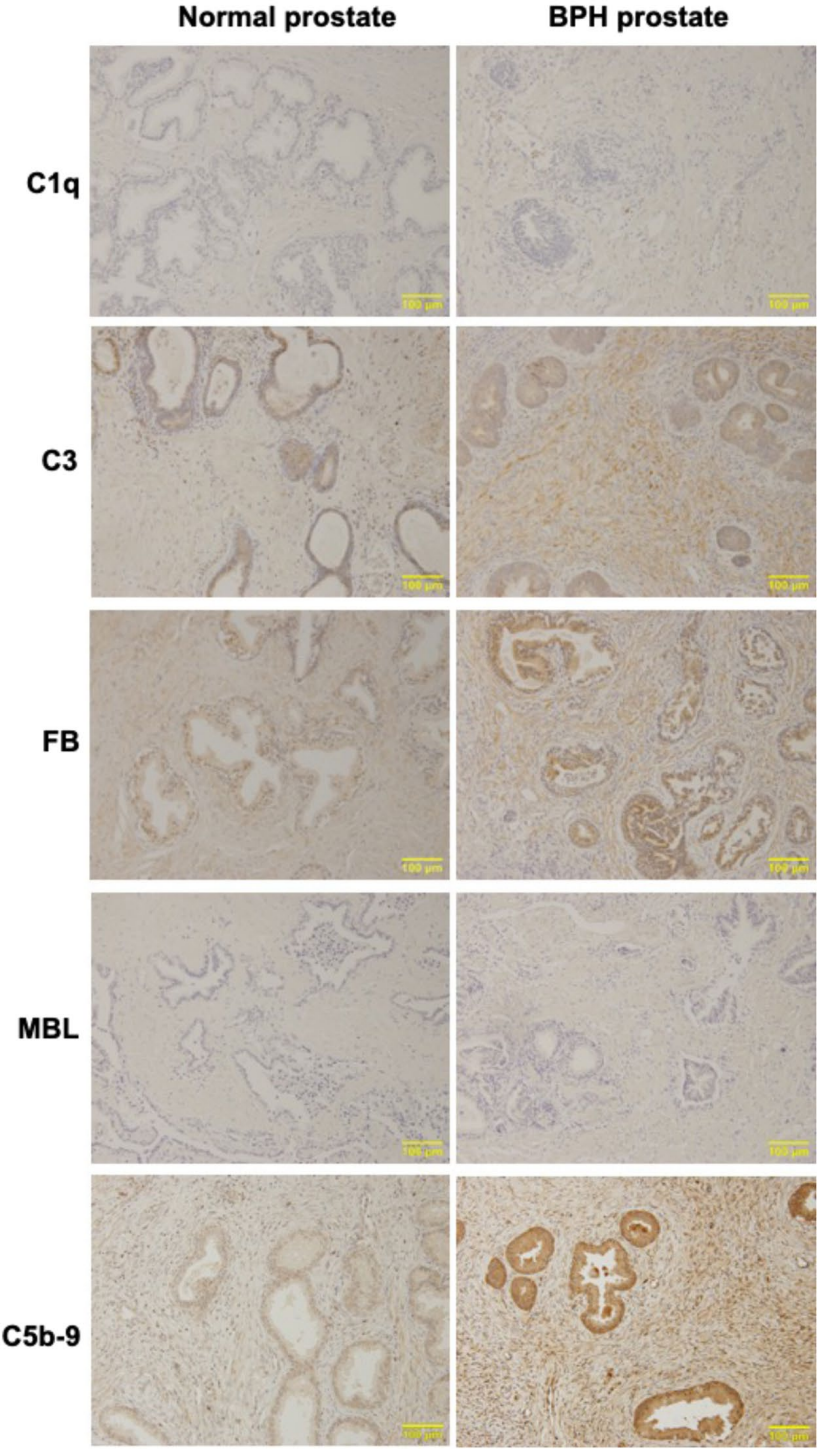
Deposition of complement factors in prostate tissues of human BPH patients. Paraffin-embedded sections of human prostate tissues obtained from BPH or bladder tumor patients (controls) were subjected to immunohistochemistry with antibodies against human complement factors. Similar results were obtained from 10 individuals for each group, and representative pictures are shown (Magnification, ×200).

To determine whether the complement system is also involved in human BPH, we performed immunohistochemical analysis of human normal and BPH prostate tissues. As shown in Fig. 3, significant expression or deposition of C3, FB and C5b-9 was observed in human BPH tissues compared to normal prostate tissues. On the other hand, there was little-to-no difference in expression or deposition levels of C1q and MBL between BPH and normal prostate tissues (Fig. 3). This suggests the involvement of the alternative pathway, and that it is unlikely that the classical and lectin pathways could be involved in the growth of human BPH.

### IgG levels in sera and localization in prostate tissues of the rat BPH model

**Figure 4:**
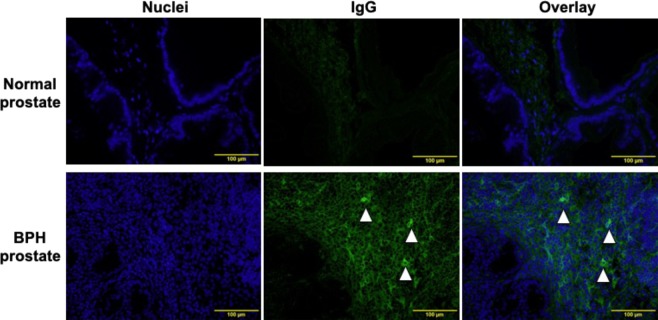
Deposition of IgG in BPH-like prostate tissues. Prostate tissues, including BPH-like and normal prostate tissues, were stained with an IgG-specific antibody and subsequently with a secondary fluorescent antibody (green fluorescence). The sections were counterstained with DAPI (blue fluorescence) (Magnification, ×400). The arrowheads indicate prostatic stromal cells that were stained with anti-rat IgG antibodies in their cytoplasm.

The results shown in Fig. 1 suggested a role for the classical pathway in the growth of BPH lesions in the rat BPH model. The classical pathway is initiated by the binding of antigen-antibody complexes to C1q protein. As shown in Fig. 4, IgG was detected sporadically in the cytoplasm of stromal cells and diffusely in the extracellular space of the stromal area in rat BPH tissue. In contrast, little-to-no IgG was detected in normal prostate tissue.

**Figure 5:**
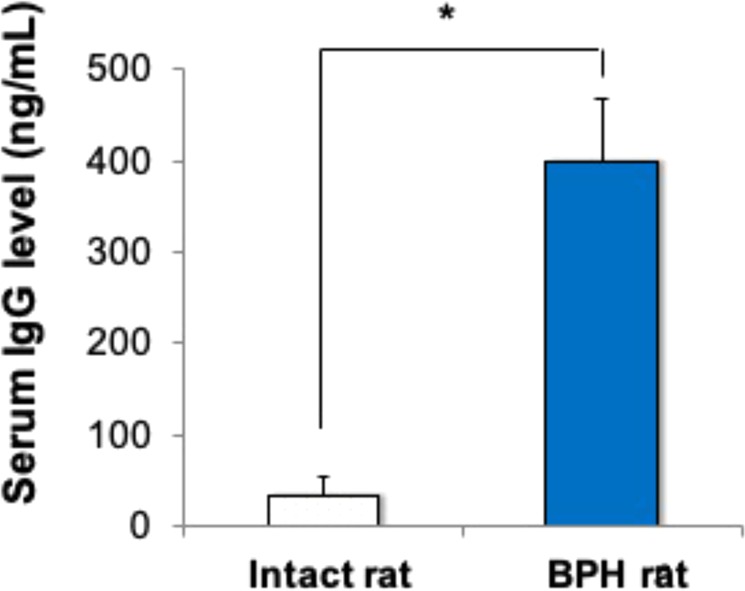
Serum IgG levels in intact rats and the rat BPH model. Serum IgG levels were measured by ELISA using sera from BPH rats 3 weeks after UGS implantation (blue bar) and from age-matched intact rats without undergoing the surgical process (white bars). Data are shown as means ± SEM (*n* = 4).

Furthermore, we measured serum IgG levels in rat sera from BPH and intact rats free from surgical UGS implantation by ELISA. Interestingly, rats that had undergone UGS implantation had significantly increased serum IgG levels compared to intact rats (398.1 ng/mL vs. 34.1 ng/mL; *p* < 0.05) (Fig. 5). These results suggest significant involvement of the classical pathway in the growth of BPH tissue in this model.

### Binding of serum IgG from the rat BPH model to normal rat prostatic stromal cells

**Figure 6:**
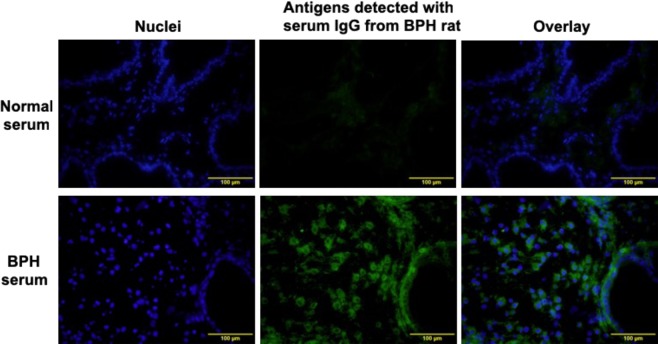
Immunofluorescence staining of prostate tissues with serum IgG from the rat BPH model. Normal prostate tissues obtained from BPH rats were incubated with sera from BPH rats at 3 weeks after UGS implantation, and bound IgG was detected with an anti-rat IgG fluorescent antibody (green fluorescence). The sections were counterstained with DAPI (blue fluorescence).

We hypothesized that autoantibodies formed antigen-antibody complexes with autoantigens in BPH tissues according to the diffuse distribution of IgG in the BPH tissues shown in Fig. 4. To confirm our hypothesis, sera obtained from BPH rats (3 weeks post-implantation) were reacted with normal prostate sections of BPH rats and bound IgG was detected by immunofluorescence staining. As shown in Fig. 6, serum IgG was strongly detected in the cytoplasm of prostatic stromal cells, while little-to-no staining was observed in the extracellular regions. In contrast, sera from intact rats that had not undergone UGS implantation showed no or slight binding of IgG to normal prostate sections (Fig. 6). These results strongly suggest that autoreactive IgG that recognizes autoantigens in rat prostatic stromal cells is produced in the rat BPH model and binds to intracellular autoantigens released into the prostatic stromal area.

### Identification of autoantigens in the rat BPH tissues recognized by IgG autoantibodies in the rat BPH model

**Figure 7:**
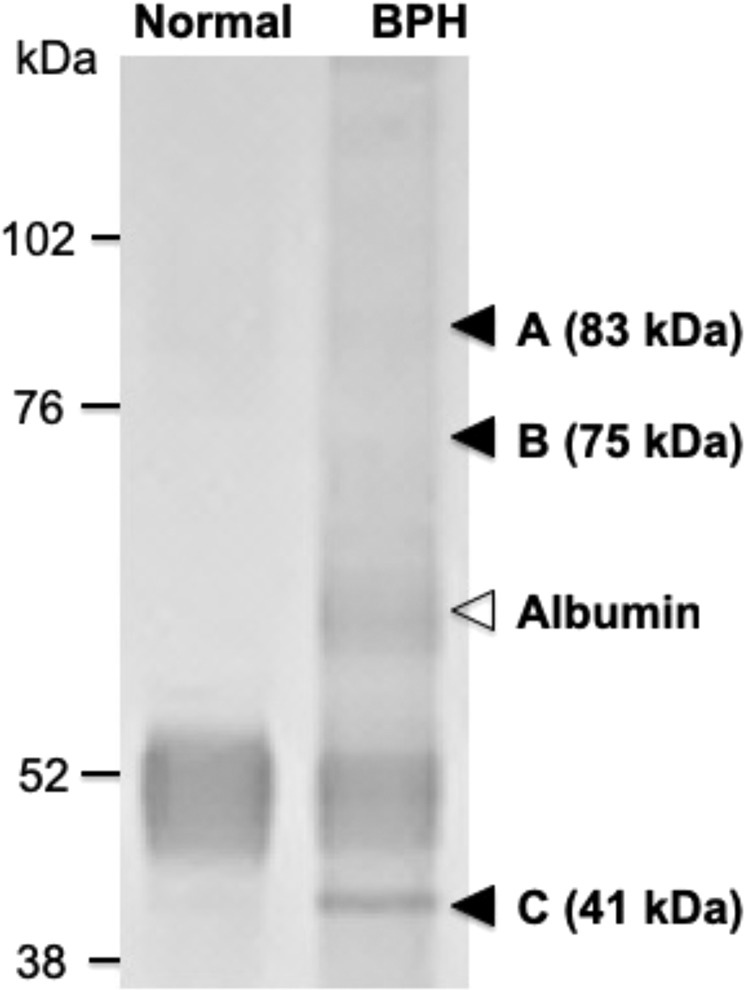
Immunoprecipitation of proteins that form a complex with autoreactive IgG in the rat BPH model. Protein extracts from normal or BPH prostate tissues in the rat BPH model were subjected to immunoprecipitation with anti-rat IgG antibody-immobilized protein G magnetic beads. The immunoprecipitated proteins were separated by SDS-PAGE and visualized by silver staining. Three protein bands with molecular masses of 83, 75, and 41 kDa were detected specifically in the extract from the BPH tissue, as indicated by arrowheads.

To identify the autoantigens in rat prostate tissues, we attempted immunoprecipitation using protein extracts from BPH and normal prostate tissues to isolate antigen-antibody complexes composed of IgG deposited on the rat BPH tissues. As shown in Fig. 7, 41, 75, and 83-kDa protein bands were detected at high levels in the BPH tissues, but were not detected in controls. According to the results of mass spectrometry and database analyses, the amino acid sequences of the immunoprecipitated proteins were identical to the following known proteins: the 41-kDa bands were α-smooth muscle actin (α-SMA) and β-actin, the 75-kDa band was annexin, and the 83-kDa band was heat shock protein 90 (Hsp90) (Supplementary Table S2).

## Discussion

To clarify the involvement of complement activation in the growth process of BPH, we analyzed the expression and deposition of complement components in prostate tissues of BPH model rats and BPH patients. In this study, we found a significant increase in the levels of complement gene expression and complement protein in the BPH tissue of the rat model compared to the corresponding normal prostate tissue. We also found significant deposition of complement protein in the prostate tissue of human BPH patients. These results suggested a significant contribution of the complement system to the growth of BPH in the rat model and BPH in human patients. The complement system consists of three distinct pathways, the classical, lectin, and alternative pathways, and their activation leads to the formation of C5b-9 and causes tissue damage. Questions raised are which complement pathway is initially activated and involved in the growth of BPH and how the complement system was activated in the prostate tissue.

**Figure 8:**
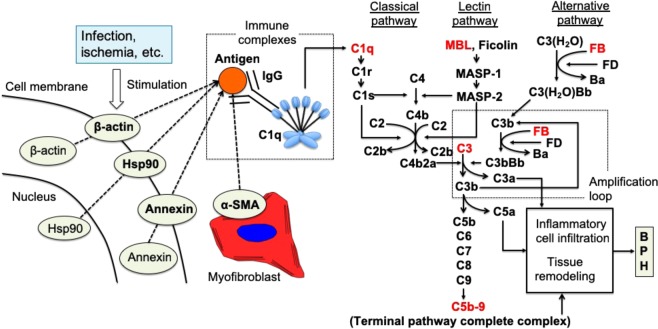
A predicted sequential model for the development of BPH-like tissue lesions through complement system activation in rat. Intracellular components of prostatic stromal cells, such as annexin, Hsp90, α-SMA, and β-actin, are exposed to the exterior of the cells likely due to infection, ischemia, or some other extracellular stimuli. Autoantibodies against the externalized components are developed under certain conditions, although the development mechanism is unclear, and antigen-antibody complexes are formed with the exposed intracellular autoantigens. The classical complement pathway is activated by binding of C1q to the complexes. Subsequently, activation of the lectin and alternative pathways occurs to accelerate the cleavage of C3 and C5, leading to the formation of anaphylatoxins (C3a and C5a) and the terminal pathway complex C5b-9. Our results strongly suggest that the complement system plays a role in the development of BPH-like tissue lesions in the rat BPH model via this mechanism.

In BPH rats, gene expression and protein levels of each complement component in prostate tissue varies depending on the time after UGS implantation. Consistent with our previous observations using DNA microarray analysis^9^, gene expression levels of C1q, the initial complement component in the classical pathway, and C3 were significantly increased in BPH prostate tissue compared to normal prostate tissue. On the other hand, gene expression levels of MBL and FB, complement components of the lectin and alternative pathways, respectively, were not detected or did not show significant differences between BPH and normal prostate tissues. In parallel with the gene expression results, C1q and C3 protein levels in BPH tissue were significantly increased compared to normal prostate tissue throughout the observation period. These results suggest that there is local production of C1q and C3 in BPH tissue. In addition, the analysis of serum C3 levels indicated that C3 was not only produced locally in prostate tissues but also induced systemically. Interestingly, MBL and FB protein levels in BPH tissues were not significantly different at the early stage (2 weeks), but showed a significant increase in the middle and late stages (3 and 8 weeks) after UGS implantation. The overall results of the immunohistochemical analysis of BPH tissues were consistent with the results of the analysis of protein levels. Taken together, we conclude that the classical pathway is activated first, then the lectin and alternative pathways are subsequently activated, culminating in the formation of C5b-9 in BPH tissue of the rat model. The classical pathway is initiated via binding of the C1 complex (which consists of the C1q, C1r, and C1s molecules) through its recognition molecule, C1q, to antigen-antibody complexes (Fig. 8). Subsequently, C1s cleaves C4 and C2, leading to the formation of the C3 convertase C4b2a complex. C4b2a then cleaves C3 into the anaphylactic peptide C3a and opsonin C3b, which covalently binds to cell surfaces via a thioester bond. As shown in Fig. 8, C3b bound on cell surfaces plays a crucial role in initiating robust alternative pathway activation that results in an amplification loop, followed by production of the anaphylactic peptide C5a and formation of C5b-9. The complement system is a major component of the innate immune system involved in defending against foreign pathogens, via complement fragments (i.e., C3a and C5a) that participate in opsonization, chemotaxis, activation of leukocytes and through cytolysis by C5b-9 formation^15^. However, excessive or uncontrolled activation of the complement system causes tissue injury and could lead to epithelial-mesenchymal transition (EMT) or tissue remodeling, and finally boost prostatic proliferation and fibrosis as seen in prostate tissue of BPH patients^16^. Our result also suggested that activation of the lectin pathway occurs in BPH prostate tissue of the rat model. The mechanism by which MBL is deposited in prostate tissue remains unknown. However, it is possible that MBL binds to cells in the prostate tissue stressed by complement activation via the classical pathway or UGS implantation itself, as proposed in the mechanisms of ischemia-reperfusion injury and age-related macular degeneration^17,18^.

Complement deposition in prostate tissues from BPH patients showed different features compared to the rat model. Compared to normal human prostate tissue, prostate tissue of BPH patients showed significant deposition of FB, C3 and C5b-9, primarily in the stromal region; whereas, no obvious deposits of C1q and MBL were observed in BPH tissues. These results suggested an important contribution of the alternative pathway, rather than the classical and lectin pathways, in the growth of BPH. However, it is possible that the classical or lectin complement pathways were activated in the early stage of BPH. In this study, the majority of prostate tissue samples were derived from patients with advanced BPH who required surgery, and histopathologic examination revealed abundant fibrous tissue in the prostate. Therefore, we hypothesized that the activation of the classical or lectin complement pathways was already quenched in the tissues of BPH patients analyzed here, however, the alternative complement pathway remained activated in advanced BPH tissues. The characteristics of the late stage (8 weeks after UGS implantation) BPH rat model are similar to those of patients with advanced BPH and support our hypothesis. On the other hand, in the previous study, the complement activation in skin carcinogenesis model mice promotes tumor growth^19^, whereas the activation of the classical and alternative pathways in the patients with some cancers is associated with a good prognosis^14^. Therefore, it is considered that the role of complement activation in various disease differs depending on the species.

Interestingly, the difference of the expression levels of some complement components between western blotting and immunohistochemical analysis was shown in BPH model rats at 8 weeks after UGS implantation (Figs. 1b and 2). Western blotting analysis showed that the protein levels of some complement components was still high in BPH tissues at 8 weeks, whereas immunohistochemical analysis showed the lower expression of these complement components. In immunohistochemical analysis of BPH tissues especially at 8 weeks, some regions are abundant in cellular components while others are abundant in fibrous components. These heterogeneity of BPH tissues have the possibilities to show these differences of complement expression pattern.

Our study in the BPH rat model suggested that complement activation in the prostate tissues is initiated via the classical pathway. The classical pathway is initiated by either IgM or IgG antigen-antibody complexes, C-reactive proteins, viral proteins, polyanions, or apoptotic cells binding to C1q. In UGS-implanted rats, we found significant deposition of IgG in the stromal region of the BPH tissues as well as increased serum IgG levels compared to UGS-unimplanted rats. In addition, serum IgG from UGS-implanted rats deposited in the cytoplasmic region of stromal cells of normal prostate tissue, while serum IgG from UGS-unimplanted rats did not. From these results, we speculated that activation of the classical pathway was initiated by deposition of IgG that recognized autoantigens in UGS-implanted rat BPH tissues.

Lastly, we searched for autoantigens in the rat BPH tissue and identified annexin, Hsp90, α-SMA, and β-actin as autoantigens recognized by IgG autoantibodies in the rat BPH model. Annexins are a family of proteins that bind calcium and phospholipid and are widely expressed, with almost all cells expressing at least one isotype of annexin^20^. Annexin identified in our study might be annexin A6 considering this molecular weight, 75 kDa^21^. Hsp90 is a molecular chaperone that is present in prostate tissues in a bound state with androgen receptors^22^. α-SMA and β-actin are proteins that are cytoskeletal constituents of smooth muscle. The prostate is an organ that has abundant smooth muscle, which is responsible for dynamic obstruction in BPH^23^. Thus, these molecules are present in various cells of normal prostate tissues. Moreover, previous studies demonstrated that the exposure of autoantigens induced by hypoxic conditions is associated with the pathophysiology of ischemic-reperfusion injury^24,25^. Therefore, these molecules are expected to function as autoantigens, with their exposure as antigens attributable to various stimuli such as hypoxia, infection, or ischemia. In addition, annexin A2 and Hsp90 have been reported to be associated with the development of prostate cancer by promoting the growth process^26–29^. These reports indicate that annexin and Hsp90 may be associated with the BPH growth process in an as yet unknown manner. In recent years, myofibroblasts expressing α-SMA on the surface of cell membranes were reported to be associated with the development of BPH and to induce prostatic proliferation^30^. These reports support the association between the four molecules identified herein and prostate growth processes (Fig. 8).

Our study has a limitation that warrants discussion. Generally, although it is reported that activation of the complement system is associated with the development of various diseases including idiopathic pulmonary fibrosis and ischemic cardiac disease^31–33^, the mechanism by which complement activation promotes inflammation and prostate growth processes has remained unclear in our study. To elucidate this mechanism, further studies by using the genetically modified rat or selective complement inhibitors are required. However, the deficiency of C1q as the activator of classical complement pathway was reported to be associated with a severe form of systemic lupus erythematosus^34^. Therefore, considering the complement activation mechanism in our study, the study using the rat model with deficiency of a complement component in alternative pathway is appropriate. Further study needed by creating the knockout rat model.

Our study indicates that the complement system is activated in BPH rat tissue and BPH patients, and may contribute to the progression of prostatic proliferation and fibrosis. In the BPH rat model, it is suggested that activation of the complement system is initiated via the classical pathway by formation of antigen-antibody complexes in prostate tissue. We identified four possible intracellular autoantigens, annexin, Hsp90, α-SMA, and β-actin, which could form antigen-antibody complexes in the prostate tissue of the rat BPH model. Our study is the first report showing a significant association between complement activation and the growth of BPH lesions in both human patients and a rat model. These findings may lead to the development of novel therapeutic agents for BPH targeted to the complement system.

## Methods

### Benign prostatic hyperplasia model rat

A previously established experimental BPH model rat with pathologically stromal component-dominant hyperplasia^9–11^ was used in this study. This BPH model rat is the model that create proliferated BPH tissues from UGS tissues, and considered to be the model rats that mimic the growth process of BPH. Pregnant female rats at 20 days of pregnancy and 7-week-old male Sprague-Dawley rats (Charles River, Kanagawa, Japan) were used to produce the BPH model rats. Briefly, the rats were anesthetized with ketamine and xylazine (80 mg/kg and 10 mg/kg intraperitoneally, respectively). First, 20-day-old male rat embryos were isolated from pregnant female rats. Second, the UGS isolated from the 20-day-old male rat embryos was implanted under the right ventral prostate of 7-week-old male rats. After the host rats had been sacrificed at 2, 3, or 8 weeks after UGS implantation, the right ventral prostate was used as BPH tissue, and the left ventral prostate tissue was used as a control. BPH model rats at 2, 3, or 8 weeks after UGS implantation were used to evaluate time-dependent histological change during growth process of BPH in this study. These stromal-dominant BPH model rats have the characteristics to increase fibrous components time-dependently (from 3 to 8 weeks) as well as human BPH tissues in previous study^10^. the All animal experiments in the present study were performed in accordance with the Guidelines for the Care and Use of Experimental Animals and protocols approved by the Animal Care Committee of Fukushima Medical University (#28058).

### Human prostate tissues

Human BPH tissues were obtained from 10 BPH patients that had undergone transurethral prostatic resection or suprapubic subcapsular prostatectomy or prostate needle-biopsy. Informed consent was obtained from all of the patients before the study (mean age 74.9 ± 6.4 years) (Supplementary Table S3). BPH was comprehensively diagnosed based on the International Prostatic Symptom Score (IPSS), digital rectal examination, ultrasonography, and prostate needle biopsy. As a control group, normal prostate tissue was obtained from 10 bladder tumor patients that had undergone radical cystectomy (mean age 70.6 ± 3.6 years). The normal prostate tissues were confirmed to have no cancer histologically by clinical pathologists. The use of human tissues in this study was approved by the Ethics Committee of Fukushima Medical University (#2585) and was performed in conformity to the provisions of the Declaration of Helsinki.

### Quantitative real-time reverse-transcription polymerase chain reaction (qRT-PCR)

Specific gene expressions in BPH tissues and normal prostate tissues were measured by quantitative real-time reverse-transcription polymerase chain reaction (qRT-PCR). RNA isolation and complementary DNA (cDNA) synthesis were performed using the ISOGEN system (Nippon Gene, Tokyo, Japan) and the SuperScript® III First-Strand Synthesis System (Thermo Fisher Scientific, Waltham, MA, USA), respectively, according to the manufacturer’s instructions. qRT-PCR was performed using the Taqman Universal PCR Master Mix (Thermo Fisher Scientific) and the StepOne^TM^ real-time PCR system, version 2.1 (Applied Biosystems, Foster City, CA, USA) according to the manufacturer’s instructions. β-actin was used as an internal control. Values for expression levels of target genes are standardized against that of β-actin gene.

### Western blotting

Protein was extracted from frozen prostate tissues in RIPA Lysis and Extraction buffer (Thermo Fisher Scientific) and subsequently subjected to SDS-PAGE. Rabbit polyclonal anti-C1q (ab171566), C3 (ab11887), and FB (ab192577) antibodies and mouse monoclonal anti-MBL (ab23461) and C5b-9 (ab83076) antibodies were purchased from Abcam (Cambridge, UK) and used in the experiment as primary antibodies. The appropriate horseradish peroxidase (HRP)-conjugated secondary antibodies were used. Protein bands were visualized with ECL^TM^ Advance Western Detection Reagent (GE Healthcare, Buckinghamshire, UK) and imaged with a ChemiDoc^TM^ XRS Plus System (BIO-RAD, Hercules, CA, USA). The membranes were re-probed with a rabbit anti-β-actin polyclonal antibody (CS 4967, Cell Signaling Laboratory, Danvers, MA, USA) and an appropriate secondary antibody to verify that protein loading was similar among the samples.

### Immunohistochemical staining

Paraffin-embedded sections of rat and human prostate tissue were prepared and incubated with anti-C1q (ab171566 for rat, ab11861 for human), C3 (ab11887 for rat, ab129945 for human), MBL (ab23461 for rat and human), FB (ab192577 for rat and human), and C5b-9 (ab83076 for rat and human) primary antibodies (Abcam). Subsequently, the sections were incubated with appropriate biotinylated secondary antibodies. Staining was detected using a streptavidin-biotin kit (Nichirei, Tokyo, Japan.

### Enzyme-linked immunosorbent assay (ELISA)

Concentrations of C3 and IgG in rat serum were measured using a Complement C3 Rat ELISA kit (ab157731, Abcam) and a Rat IgG ELISA Kit SimpleStep (ab189578, Abcam) according to the manufacturer’s instructions. Sera from the rat BPH models at 3 weeks after UGS implantation were used in the experiments. Age-matched intact rats that had not undergone surgical implantation were used to obtain the intact serum samples. Absorbance was measured at 450 nm using a Varioskan Flash microplate reader (Thermo Fisher Scientific).

### Immunofluorescence staining

For staining of IgG deposited in prostate tissues, sections from frozen BPH and normal prostate were directly used for immunofluorescence staining. Prior to antibody staining, separate sections from frozen normal prostate were incubated with sera from a BPH rat at 3 weeks post-UGS implantation or from intact rats. Those sections were incubated with a rabbit anti-rat IgG primary antibody (ab102168, Abcam). Alexa Fluor 488-conjugated goat anti-rabbit IgG antibodies (ab150081, Abcam) were added to the sections followed by incubation. Stained sections were mounted with Vectashield mounting medium with DAPI (Vector Laboratories, Burlingame, CA, USA). Images were obtained using a fluorescence microscope OLYMPUS BX51 (Olympus, Tokyo, Japan) and merged using a cellSens photo-editing software (Olympus).

### Immunoprecipitation and protein identification by mass spectrometry

Immunoprecipitation was performed using PureProteome Protein G Magnetic Beads (Merck Millipore, Darmstadt, Germany) and the capture antibody, goat anti-rat IgG Fc (ab97086, Abcam), according to the manufacturer’s instructions. The bound immune complexes were analyzed by SDS-PAGE under reducing conditions. Protein bands were visualized by silver staining. Individual stained bands were excised from gels, destained, and subjected to enzyme digestion as described previously^25^. The peptides were separated using a trypsin/lysyl endopeptidase solution (150 ng/30 μL), and amino acid sequences were determined by mass spectrometry (Easy-nLC 1000/Orbitrap Elite, Thermo Fisher Scientific). Mass spectrometry data were processed and subjected to database searches using the Mascot server (Matrix Science, Boston, MA, USA) or Sequest HT (Thermo Fisher Scientific).

### Statistical analysis

Statistical analyses were performed using SPSS Statistics 21 (IBM, Armonk, NY, USA). All values are shown as means ± SEM. Data were analyzed using the unpaired Student’s *t-*test. Statistical significance was defined as *p* < 0.05.

## Supplementary information

Supplementary Information.
