# Supplementary material for: Complement activation by autoantigen recognition in the growth process of benign prostatic hyperplasia
Source: Sci Rep. 2019 Dec 30;9:20357. doi: 10.1038/s41598-019-57001-w (PMC6937285; doi:10.1038/s41598-019-57001-w)
Supplement: Supplementary file 1 — Supplementary Information. [file 41598_2019_57001_MOESM1_ESM.pdf]

Supplementary Information for

Complement activation by autoantigen recognition in the growth process of benign prostatic hyperplasia

Junya Hata[1], Takeshi Machida[2], Kanako Matsuoka[1], Seiji Hoshi[1], Hidenori Akaihata[1], Hiroyuki Hiraki[1], Toshiyuki Suzuki[3], Soichiro Ogawa[1], Masao Kataoka[1], Nobuhiro Haga[1], Kei Ishibashi[1], Yoshimi Homma[3], Hideharu Sekine[2], and Yoshiyuki Kojima[1]

Correspondence should be addressed to J.H (E-mail: akju826@fmu.ac.jp).

Supplementary information includes:

Table S1 to S3 and Fig. S1

Supplementary Table S1. Genes for complement system notably upregulated or downregulated in BPH tissues of the rat BPH models compared to their normal prostate tissues.

Probe set	Gene symbol	Description	Genbank	Average fold change (Min to Max, n=4)	t-test P-value
1367800_at	*Plat*	plasminogen activator, tissue	NM_013151	4.011 (2.433 to 5.897)	$9.9x10^{-3}$
1370215_at	*C1qb*	complement component 1, q subcomponent, beta polypeptide	AW434057	3.373 (0.972 to 32.2)	$5.6x10^{-3}$
1383391_a_at	*C2*	complement component 2	AI716125	2.733 (0.268 to 37.1)	$3.9x10^{-3}$
1373025_at	*C1qc*	complement component 1, q subcomponent, gamma polypeptide	AI411618	2.424 (0.365 to 30.39)	$2.8x10^{-3}$
1376652_at	*C1qa*	complement component 1, q subcomponent, alpha polypeptide	BF418957	2.957 (0.566 to 34.45)	$2.5x10^{-3}$
1383241_at	*C1r*	complement component 1, r subcomponent	BI292425	2.026 (0.804 to 8.978)	$2.2x10^{-3}$
1368323_at	*Tfpi*	tissue factor pathway inhibitor	NM_017200	16.07 (5.054 to 30.01)	$5.0x10^{-4}$
1367899_at	*F2r*	coagulation factor II (thrombin) receptor	NM_012950	7.475 (3.692 to 11.24)	$3.9x10^{-4}$

Probe Set	Gene Symbol	Description	Genbank	Average fold change (Min to Max)	t-test P-value
1371037_at	*Pros1*	protein S (alpha) /// protein S (alpha)	U06230	0.491 (0.315 to 0.62)	$4.2x10^{-3}$
1367929_at	*Cd59*	CD59 antigen	NM_012925	0.456 (0.375 to 0.571)	$9.3x10^{-4}$
1387798_a_at	*Crry*	complement receptor related protein	D42115	0.372 (0.286 to 0.49)	$2.8x10^{-5}$

The expression profiles were obtained by a microarray analysis using mRNAs from BPH-like and normal prostate tissues of the rat BPH models at 3 weeks after implantation of rat embryonic urogenital sinus to the prostate tissue of pubertal male rat (Hata et al. 2016).

Supplementary Table S2. Mass spectrometry results for proteins from rat BPH tissues isolated by immunoprecipitation with anti-rat IgG antibodies.

Lane	Score	Matched protein	Mw
A	965	Heat shock protein HSP 90-beta	83,229
	617	Heat shock protein HSP 90-alpha	84,762
B	*1	Annexin	75,706
C	2225	Actin, cytoplasmic 1 (β-actin)	41,710
	1420	Actin, aortic smooth muscle (α-SMA)	41,982

Supplementary Table S3. Patients characteristics

	Normal	BPH	P-value
n	10	10	-
Age (years)	70.6±3.6	74.9±6.4	0.10
Prostate volume (ml)	24.4±13.7	58.9±34.8	<0.01
PSA (ng/ml)	2.2±1.5	4.7±4.2	0.16
IPSS (total score)	8.3±4.7	14.5±7.5	0.13
QOL index	4.1±1.5	4.6±1.2	0.87
OABSS	5.1±2.1	4.1±2.3	0.22

PSA, prostatic specific antigen; IPSS, International Prostate Symptom Score;

QOL, quality of life; OABSS, Overactive Bladder Symptom Score

Supplementary Fig. S1.

C1q

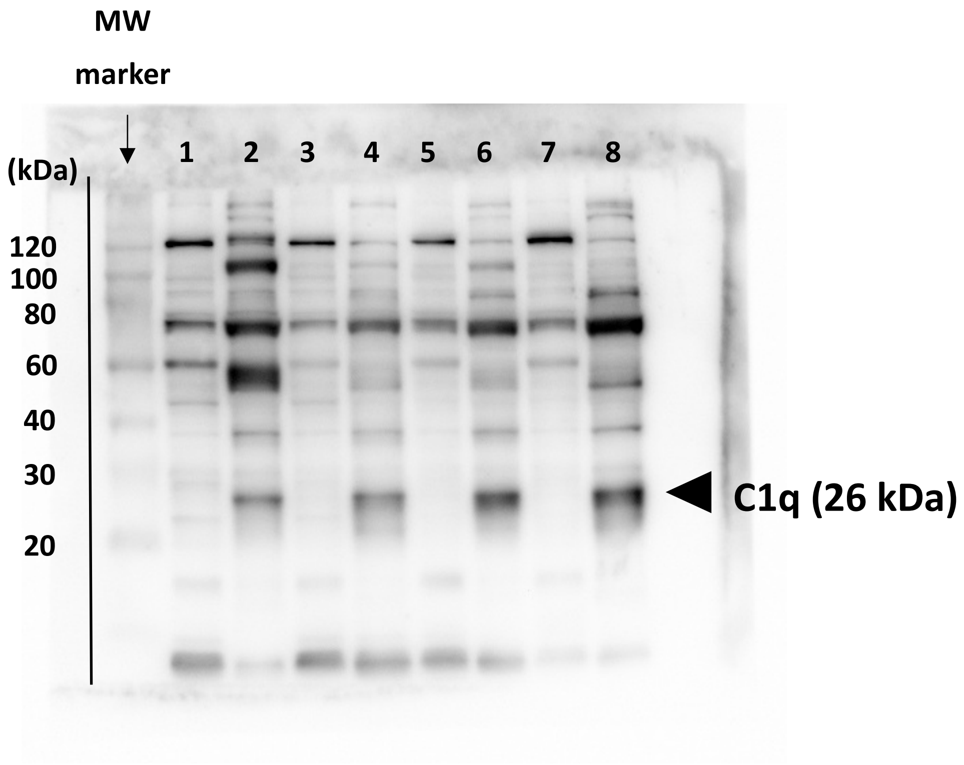

β-actin

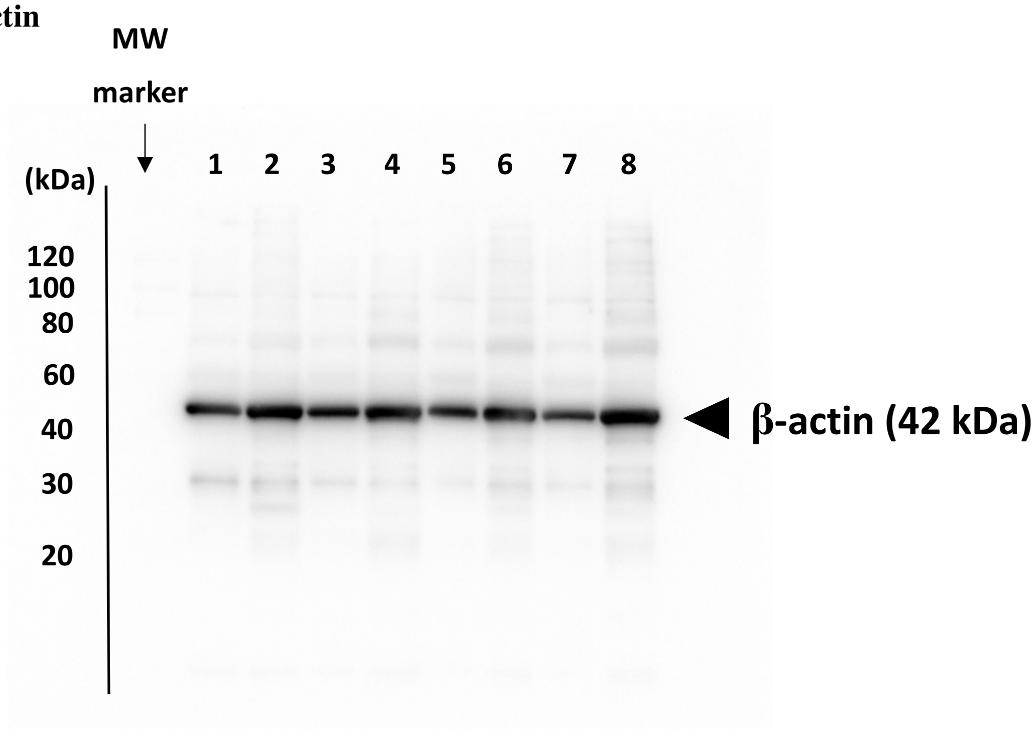

C3

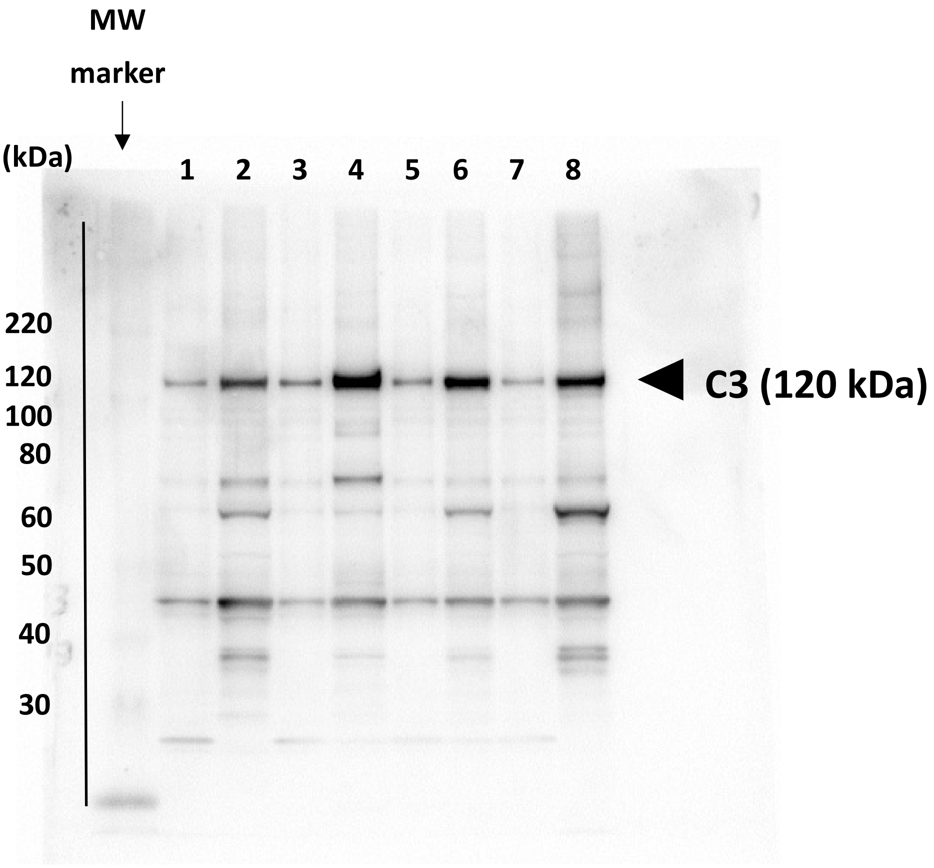
MW
marker
(kDa)
1 2 3 4 5 6 7 8
220
120
100
80
60
50
40
30
C3 (120 kDa)

β-actin

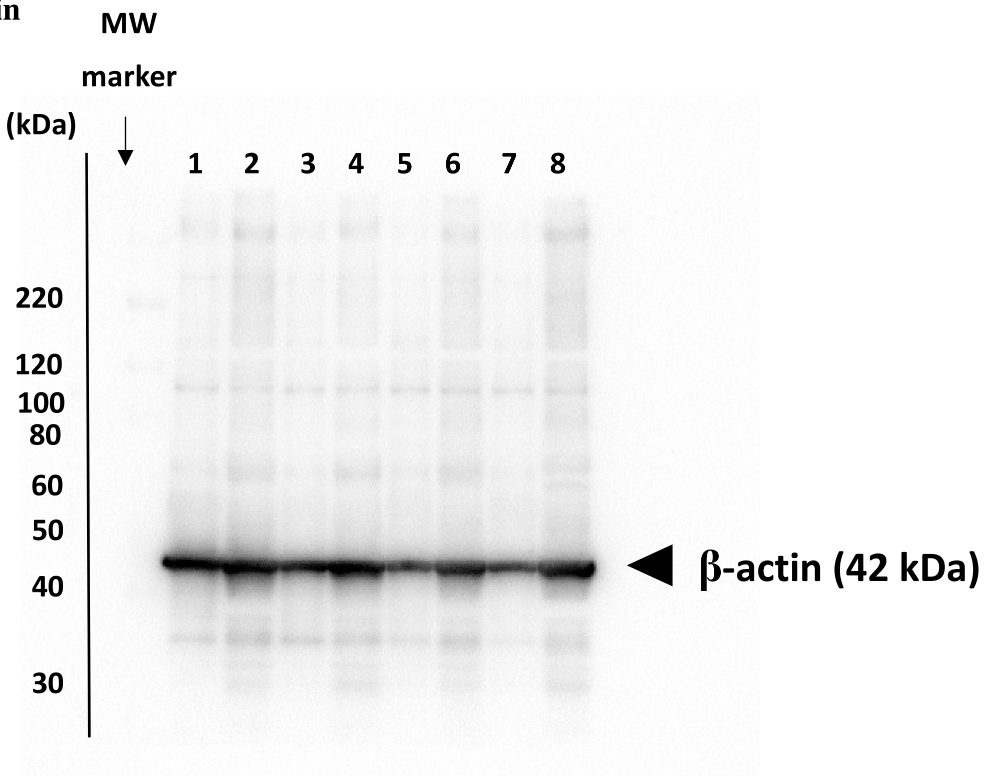
MW
marker
(kDa)
1 2 3 4 5 6 7 8
220
120
100
80
60
50
40
30
β-actin (42 kDa)

FB

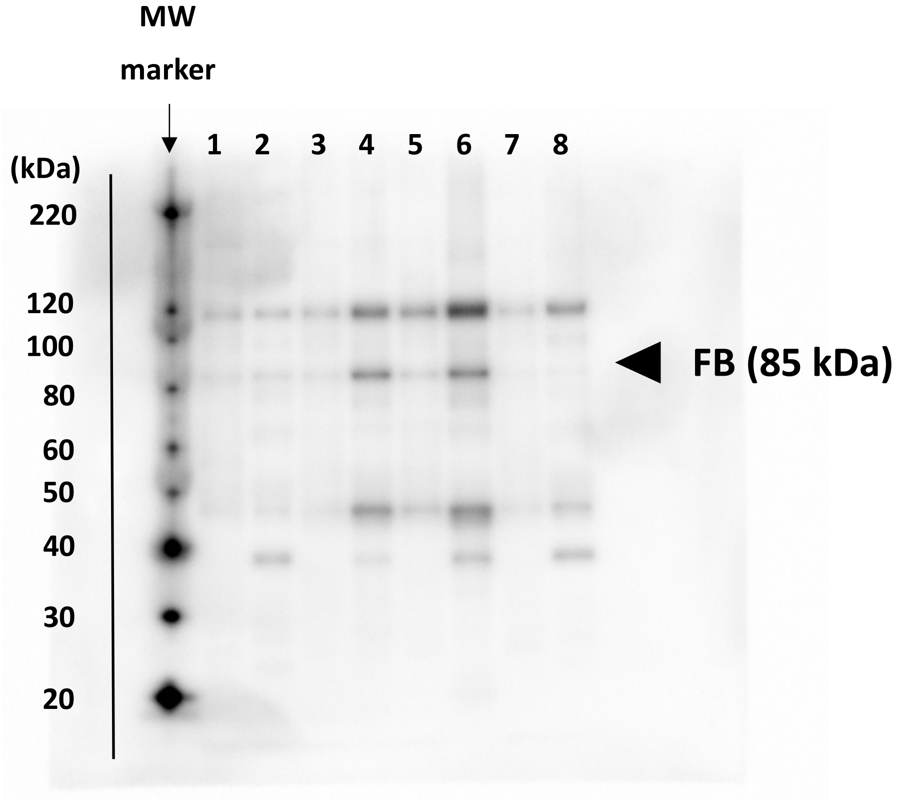
MW
marker
(kDa)
1 2 3 4 5 6 7 8
220
120
100
80
60
50
40
30
20
FB (85 kDa)

β-actin

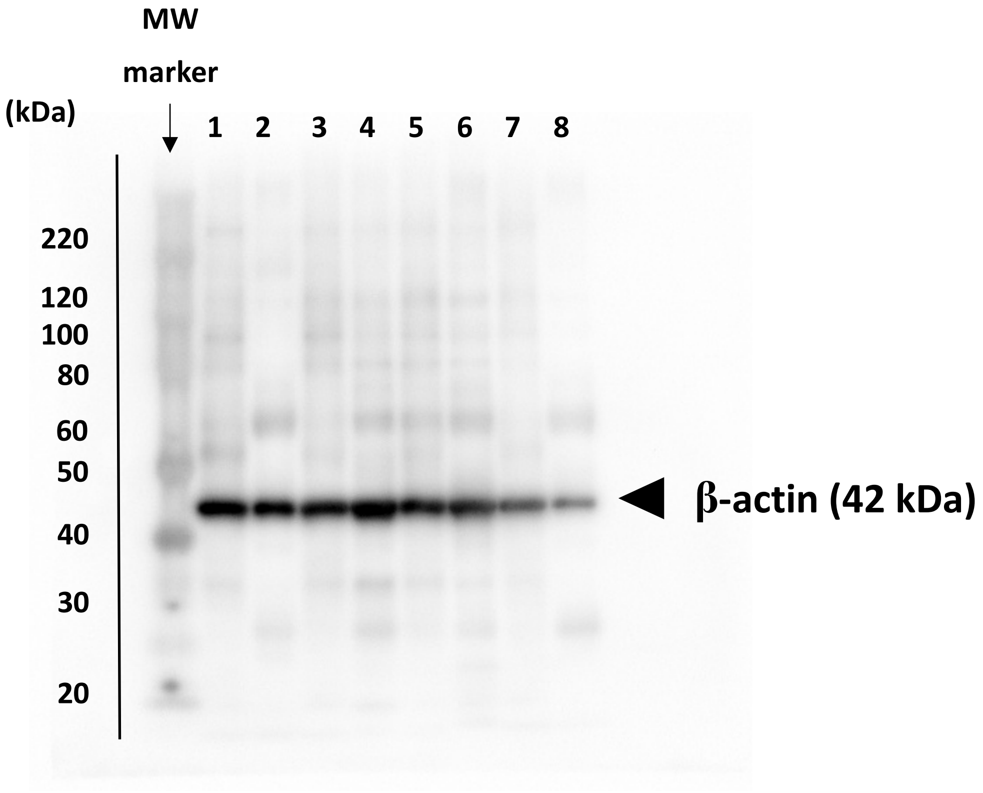
MW
marker
(kDa)
1 2 3 4 5 6 7 8
220
120
100
80
60
50
40
30
20
β-actin (42 kDa)

MBL

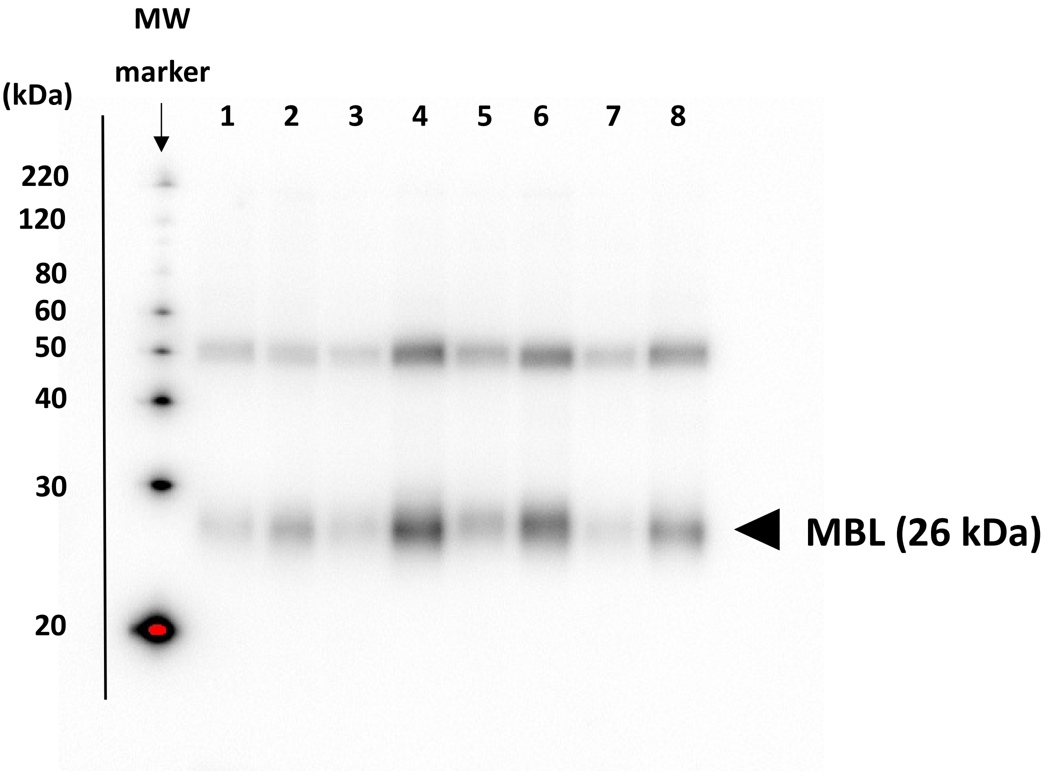
MW
marker
(kDa)
1 2 3 4 5 6 7 8
220
120
80
60
50
40
30
20
MBL (26 kDa)

β-actin

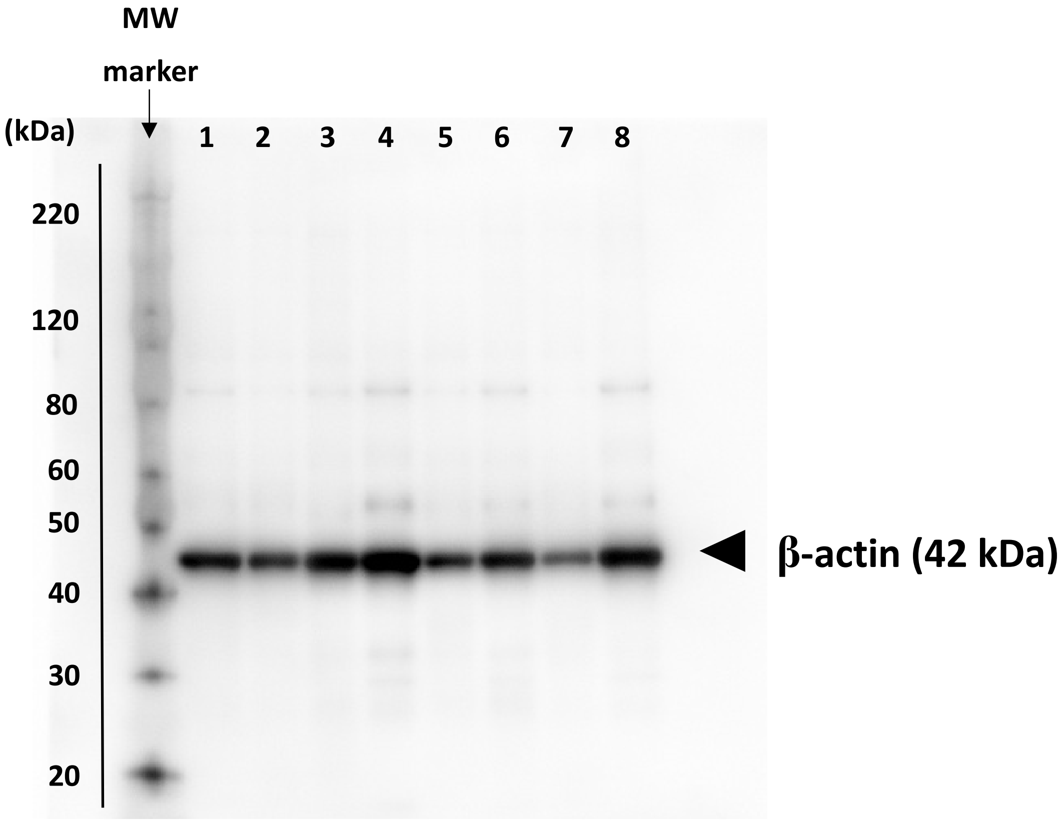
MW
marker
(kDa)
1 2 3 4 5 6 7 8
220
120
80
60
50
40
30
20
β-actin (42 kDa)

C5b-9

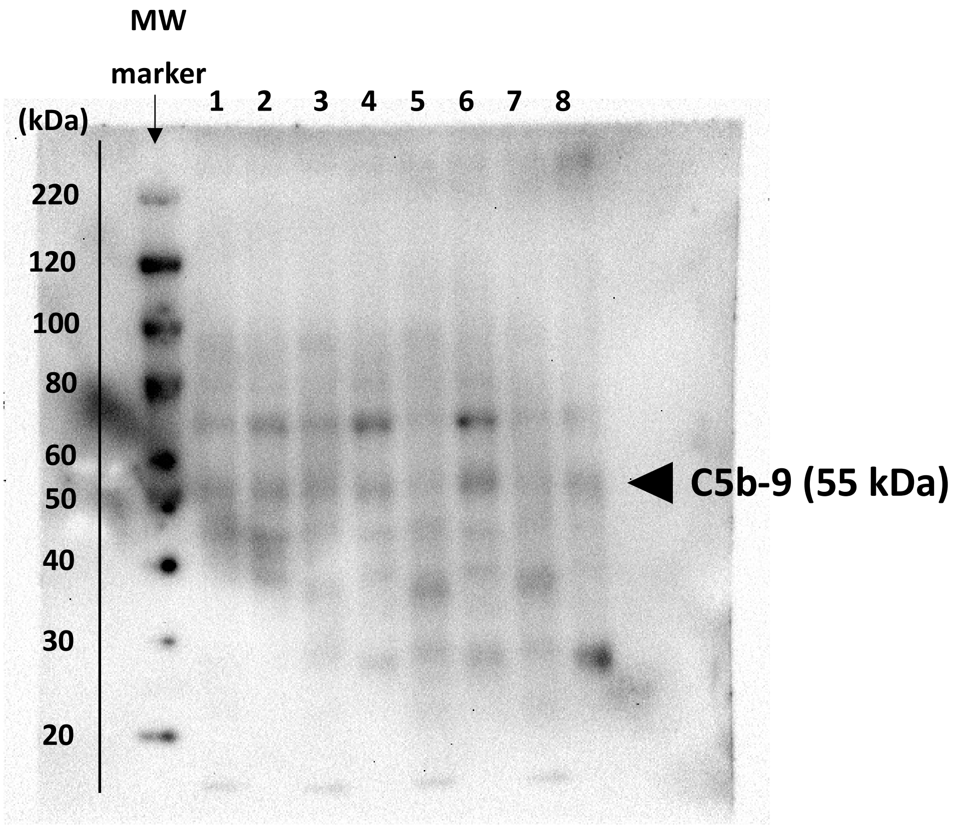
MW
marker
(kDa)
1 2 3 4 5 6 7 8
220
120
100
80
60
50
40
30
20
C5b-9 (55 kDa)

β-actin

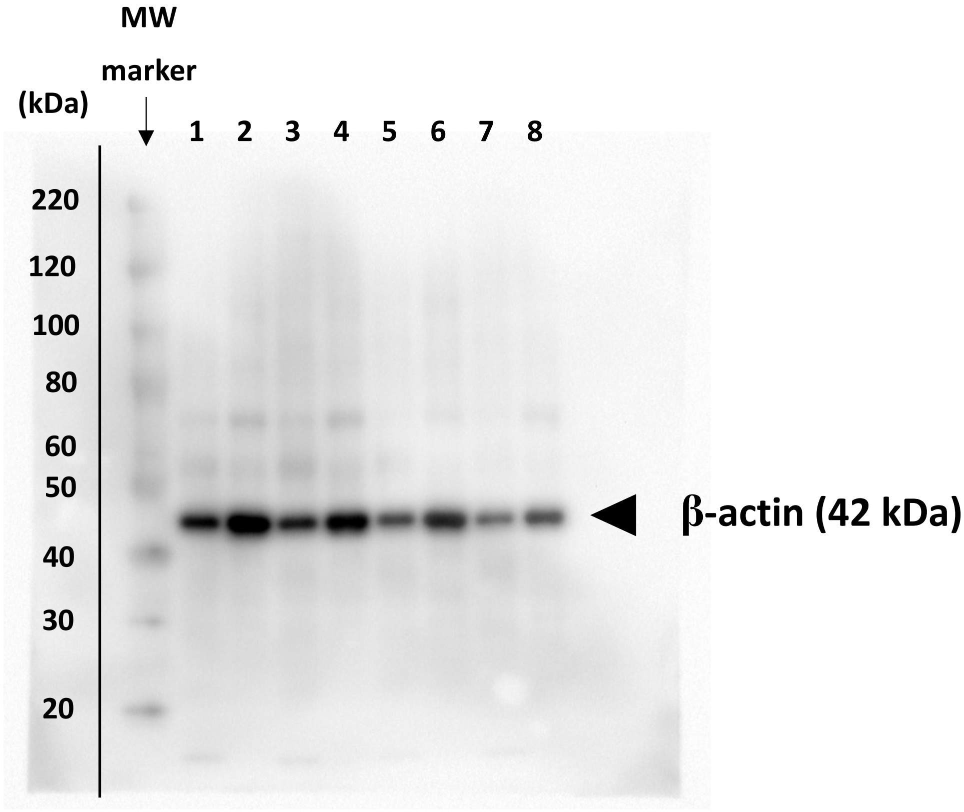
MW
marker
(kDa)
1 2 3 4 5 6 7 8
220
120
100
80
60
50
40
30
20
β-actin (42 kDa)

Supplementary Fig. S1. Full-size gel detected for western blotting shown in the main figure.

Uncropped western blotting analysis is shown. The Leftmost lane indicates molecular weight (MW) marker. The lane 1 and 2 indicate normal prostate tissues and BPH tissues at 2 weeks after UGS implantation, respectively. The lane 3 and 4 indicate normal prostate tissues and BPH tissues at 3 weeks after UGS implantation. The lane 5 and 6 indicate normal prostate tissues and BPH tissues at 8 weeks after UGS implantation.

Supplementary Fig. S2

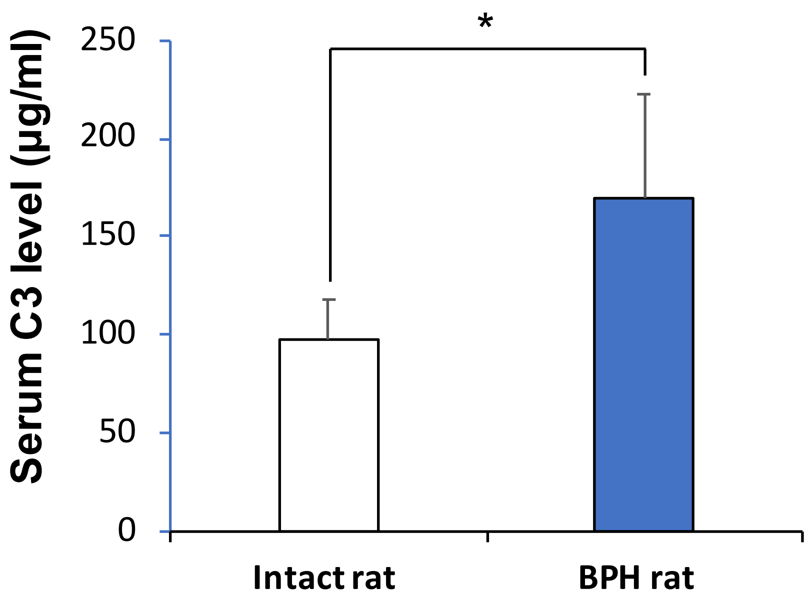

Supplementary Fig. S2. Serum C3 levels in intact rats and the rat BPH model.

Serum C3 levels were measured by ELISA using sera from BPH rats 3 weeks after UGS implantation (blue bar) and from age-matched intact rats without undergoing the surgical process (white bars). Data are shown as means ± SEM ($n = 4$).

Supplementary Fig. S3.

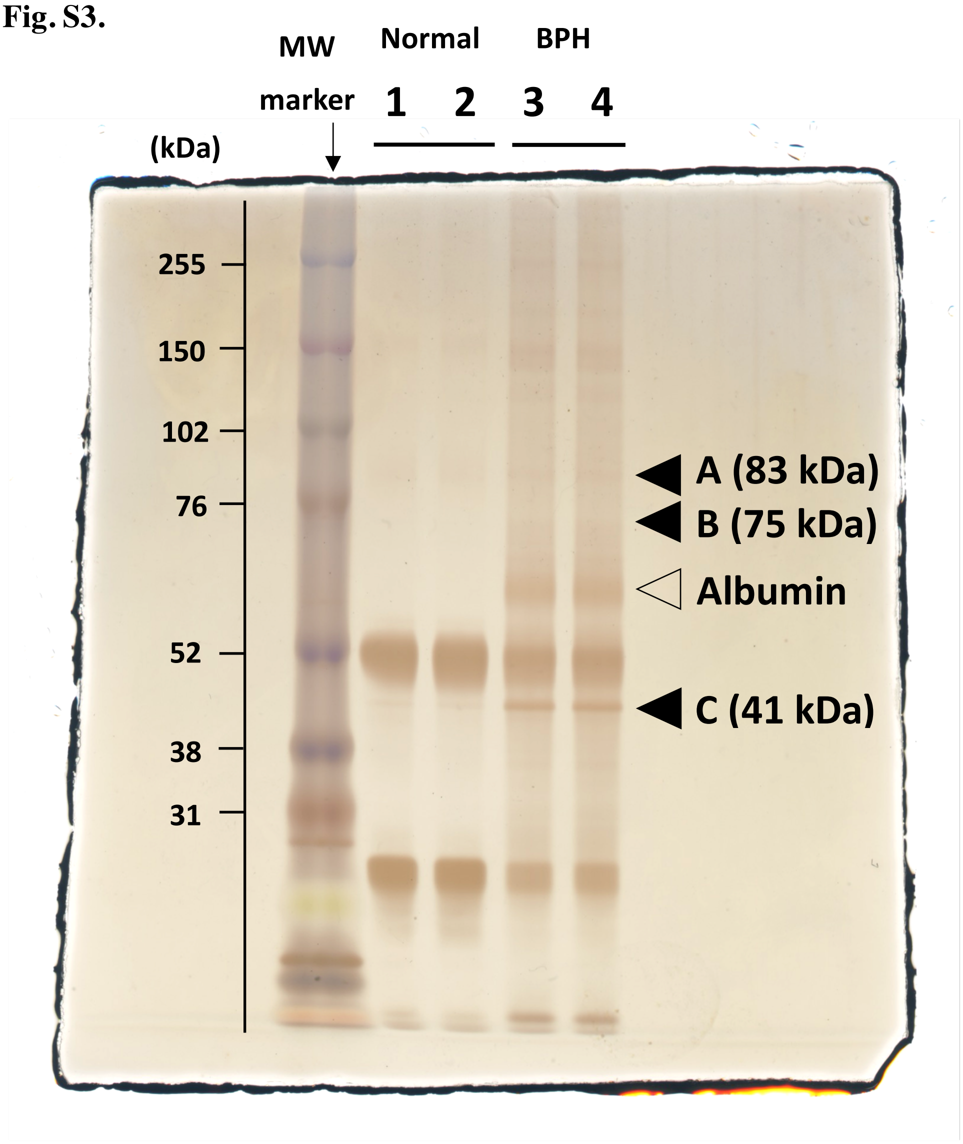

Supplementary Fig. S3. Full-size gel detected for immunoprecipitation shown in the main figure.

Uncropped immunoprecipitation analysis visualized by silver staining is shown. The Leftmost lane indicates molecular weight (MW) marker. The lane 1 and 2 indicate normal prostate tissues immunoprecipitated of the same samples. The lane 3 and 4 indicate BPH tissues immunoprecipitated of the same samples.
